# Staff acceptability and patient usability of a self-screening kiosk for atrial fibrillation in general practice waiting rooms

**DOI:** 10.1016/j.cvdhj.2022.07.073

**Published:** 2022-08-04

**Authors:** Kirsty McKenzie, Nicole Lowres, Jessica Orchard, Charlotte Hespe, Ben Freedman, Katrina Giskes

**Affiliations:** ∗ Heart Research Institute, The University of Sydney, Sydney, Australia; † Faculty of Medicine and Health and Charles Perkins Centre, University of Sydney, Sydney, Australia; ‡ Agnes Ginges Centre for Molecular Cardiology, Centenary Institute, Sydney, Australia; § Department of General Practice, School of Medicine, University of Notre Dame, Sydney, Australia

**Keywords:** Atrial fibrillation, General practice, Screening, Self-screening, Stroke prevention, Self-service kiosks, Older people

## Abstract

**Background:**

Current Australian and European guidelines recommend opportunistic screening for atrial fibrillation (AF) among patients ≥65 years, but general practitioners (GPs) report time constraints as a major barrier to achieving this. Patient self-screening stations in GP waiting rooms may increase screening rates and case detection of AF, but the acceptability of patient self-screening from the practice staff perspective, and the usability by patients, is unknown.

**Objective:**

To determine staff perspectives on AF self-screening stations and factors impacting acceptability, usability by patients, and sustainability.

**Methods:**

We performed semi-structured interviews with 20 general practice staff and observations of 22 patients while they were undertaking self-screening. Interviews were coded and data analyzed using an iterative thematic analysis approach.

**Results:**

GPs indicated high levels of acceptance of self-screening, and reported little impact on their workflow. Reception staff recognized the importance of screening for AF, but reported significant impacts on their workflow because some patients were unable to perform screening without assistance. Patient observations corroborated these findings and suggested some potential ways to improve usability.

**Conclusion:**

AF self-screening in GP waiting rooms may be a viable method to increase opportunistic screening by GPs, but the impacts on reception workflow need to be mitigated for the method to be upscaled for more widespread screening. Furthermore, more age-appropriate station design may increase patient usability and thereby also reduce impact on reception workflow.

**Key Findings**

• General practitioners were positive about atrial fibrillation (AF) self-screening and reported minimal impacts on their workflow.
• Receptionists and practice managers appreciated the importance of AF screening; however, the self-screening kiosk increased their workload and it was not prioritized in busy periods.
• Many patients were unable to complete self-screening without assistance from reception staff.
• Further refinements are required to the AF self-screening station and its integration into the workflow of reception staff to make it sustainable for larger-scale implementation.

## Introduction

Atrial fibrillation (AF) is the most common arrhythmia in older adults,^1^ with approximately one-third of those with the condition experiencing no symptoms.^2^ Around 30 percent of strokes are caused by AF, with asymptomatic AF conferring a similar stroke risk as symptomatic disease.^2^ Screening for AF in older people is required to reduce the ∼10% of ischemic strokes related to AF that are first diagnosed at the time of stroke.^3^ Current Australian and European guidelines recommend opportunistic screening by pulse palpation or electrocardiogram (ECG) rhythm strip among adults aged 65 years and older to identify asymptomatic AF cases.^1^

General practitioners (GPs) are uniquely placed to screen and initiate management for AF. In Australia more than 90% of adults aged ≥65 years see their GP at least annually, and around 70% see their GP 2 or more times per year.^4^ However, an international survey found that Australian GPs screen only 11% of their patients aged 65 years and over.^5^ To date, interventions to improve AF screening rates in general practice have focused on the use of screening devices during doctor or nurse consultations, or by receptionists. However, time constraints have been identified as the main barrier for implementing staff-led screening.^6^, ^7^, ^8^, ^9^ To overcome time barriers, a possible approach is for patients to self-screen in GP waiting rooms prior to their consultation.^10^ A self-screening approach was trialed in 2020–2021 in the AF SELF SMART study.^10^ In this study, AF self-screening kiosks for patients ≥65 years were introduced in 6 GP waiting rooms across New South Wales, Australia. Results of the impact of self-screening on AF screening rates and case detection have been described elsewhere.^11^

Successful implementation of a self-screening program for AF would improve detection of AF and, together with guideline-recommended treatment, prevent AF-related strokes. Although improvements in screening rates and case detection are important outcomes to evaluate its potential for upscaling, the utility of the method from the GP, practice staff, and patient perspectives are important considerations. Prior to wider implementation, it is important to understand both the system- and person-level factors that need to be addressed to ensure successful upscaling of self-screening. Therefore, this study aimed to explore the acceptability, usability, and sustainability of AF self-screening in GP waiting rooms from the perspectives of general practice staff, and to identify issues pertinent to patient utilization of the self-screening station.

## Methods

### Design

This was a prospective qualitative analysis of the AF SELF SMART study^10^ using thematic analysis as described by Braun and Clarke,^12^ performed between March 2022 and October 2022. Ethics approval was granted by the Human Research Ethics Committees of the University of Sydney (Project no: 2019/382) and the 10.13039/501100001800University of Notre Dame Australia (Project no: 019145S). The study is reported according to the consolidated Criteria for Reporting Qualitative Research.^13^

### Overview of AF SELF SMART procedure

**Figure 1:**
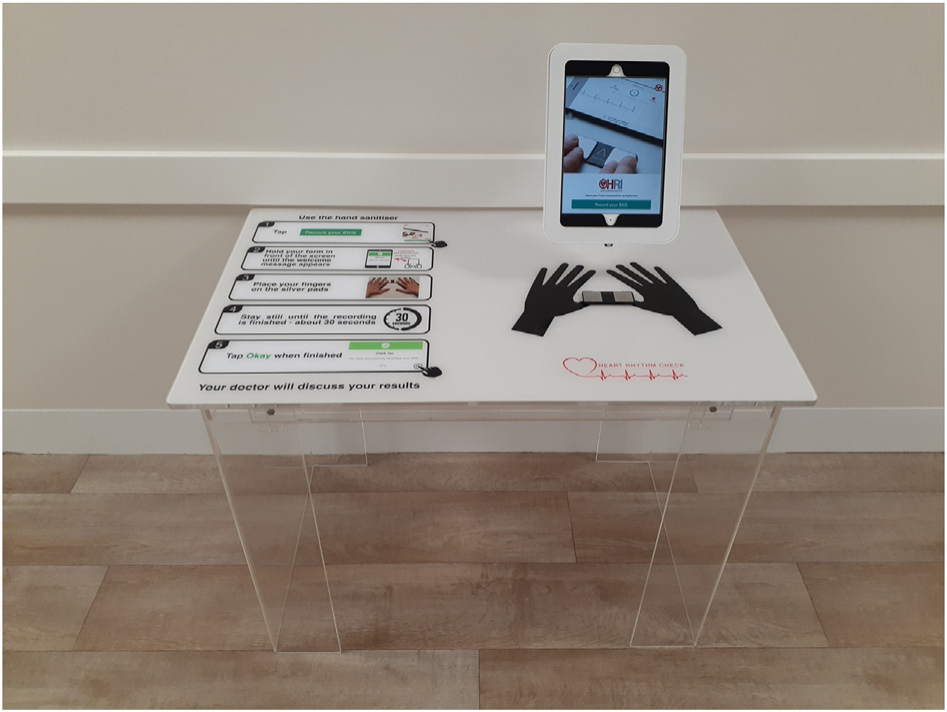
Atrial fibrillation self-screening station.

The AF SELF SMART self-screening protocol is described elsewhere.^10^ Briefly, the self-screening station included a mounted Kardia ECG device and iPad, which recorded a 30-second lead-1 ECG (Figure 1). The table was placed in the practice waiting room. Eligible patients (aged ≥65 years, no previous AF diagnosis, with a face-to-face GP appointment) were identified by the integrated software. When these patients registered with reception staff, they were given a printed QR code and directed to the self-screening station. For the “on-boarding process,” patients scanned their QR code, which auto-registered their details. They then followed prompts to record their ECG. Results of the ECG were not visible to the patient. The integrated software exported the ECG trace and algorithm interpretation to the patient’s electronic medical record, in the same manner as pathology results. GPs were able to review the screening outcome and discuss this with the patient during their consultation. All investigations and management decisions were at the discretion of the treating GP.

AF SELF SMART employed an iterative process for the design of the self-screening kiosk, which included a staged rollout of the screening stations across the 6 practices. Based on feedback from the first 4 practices, enhancements were made to the physical station setup and patient instructions, as well as the software integration, as described in Supplemental Item 1.

### Participant recruitment

Purposive sampling was used for participant recruitment. Receptionists, practice managers, and GPs from practices participating in AF SELF SMART were deemed eligible to participate, in order to capture the experiences and views of all practice staff involved in the screening process. Practice staff were invited to participate in the interviews, and those agreeing to participate provided written informed consent. All patients who consented to undertake self-screening were eligible. Patients were directly approached and invited to participate in the observations, and written informed consent was obtained.

### Data collection

A semi-structured interview guide was developed for staff interviews (Supplemental Item 2) to explore perceptions and experiences of the AF SELF SMART program, including impacts on workflow and time constraints, perceived benefits, facilitators and barriers, and factors impacting sustainability. These guides were based on our previous qualitative work (which were pilot tested with health professionals),^6^, ^7^, ^8^ consultation with general practice staff, and discussions with the multidisciplinary research team.

All interviews were conducted by researcher K.M. Across the 6 practices 20 staff were interviewed. Staff interviews averaged 10 minutes. Thirteen were conducted face-to-face and 7 via telephone owing to COVID-19. All interviews were audio-recorded, except for 2 where detailed notes were taken, as interviewees asked to not be recorded. Data collection ceased when data saturation was reached.

Across 2 practices, 22 patients were observed while they undertook self-screening and were asked questions to clarify difficulties encountered and asked for suggestions for improvement. Responses to questions were collected using detailed notes. Observation data included any ad hoc comments made by the patient during screening, their physical behavior (eg, where they were looking), and aspects that patients needed help with. These observations took approximately 5 minutes per patient.

### Data analysis

Audio-recorded data were transcribed verbatim by researcher K.M. and stored and coded in NVivo 12. Data were analyzed using an iterative thematic analysis approach described by Braun and Clarke.^12^ The analysis used both an inductive (data-driven) and a deductive (research-driven) approach to coding. Each transcript was coded line by line by K.M. based on identification of similar concepts, ideas, and patterns in the data, and with reference to the key evaluation questions. Attention was also given to whether there were any deviant cases (uncommonly expressed ideas) that might be important for the study. Once all transcripts had been coded, a second researcher (N.L.) coded 3 transcripts independently, and the 2 researchers met to compare coding. There were few discrepancies and these were addressed collaboratively. Once an initial set of codes was derived, K.M., N.L., and K.G. grouped these into themes. To ensure that the data reflected the views of participants and were not overly simplified, emerging themes were then checked against the transcripts as a whole. After the themes were finalized, the themes were developed into a narrative, which both explained the themes and provided evidence from the data in the form of quotations. Rigor was addressed by an iterative process of constant comparison to code and analyze the data (moving between codes/emerging themes and transcripts); double coding; and continual discussion of emerging themes within the team including 2 academic GPs.

## Results

The participants consisted of 20 general practice staff across 6 practices (2 urban, 2 rural, and 2 regional) and 22 patients from 2 practices (1 urban and 1 regional). The practice staff consisted of 6 GPs (3/6 female), 9 receptionists (8/9 female), and 5 practice managers (5/5 female). All patients were aged over 65 years (by nature of the study) and no other demographic data were collected for patients. Discussions of themes are organized around the key research questions. A codebook of final themes is supplied in Supplemental Item 3. Staff were identified by a participant code number, with R, PM, and GP indicating receptionist, practice manager, and general practitioner, respectively. Illustrative quotes for each theme are provided in Table 1.

**Table 1:** Major themes

Theme	Illustrative quotes (responder designation)
Acceptability of the concept	
“A good idea”	*I think it’s a good idea. Preventing strokes is a very, very good thing (GP-3)* *It’s had some benefits of identifying some patients with atrial fibrillation…and we now have a few more of them on treatment for their atrial fibrillation than we did when we started (GP-13*)
“A good idea, but…”	*I love the work that being done. It is very hard on reception. Very hard (R-2).*
Staff views on acceptability to patients	
“Patients are happy to do it”	*I think most that I’ve dealt with think it’s a great idea. And so easy, 30 seconds and the results are with the doctor as soon as they’ve done it (R-1)*
Patient refusal	*[some patients] just say no I don’t want to do that, I don’t want to know if there is something wrong (PM-12)*
Staff views on usability for patients	
Patients need assistance with screening	*I know that the system was made to be used by the patient only, without assistance from any of our staff, such as myself, but I feel like patients were not very capable of doing it on their own. So reception did have to assist them through the process (R-20)*
Some patients not “tech-savvy”	*We told them to read the instructions but some of them still just were not computer literate or they just had trouble understanding it (PM-5)* *Quite a few of them did struggle. I’m putting it down to lack of technology and experience with technology…That might come down to our location as well. Some of our patients do come from a lower socioeconomic background, so I am putting it down to that as well (R-20)*
Impact on workflow	
Minimal impact on GP workflow	*It just takes half a minute (GP-14)*
Significant impact on reception workflow	*It takes 2 minutes from standing up to sitting down again. It doesn’t sound like much but it adds up (R-9)*
Reception too busy to assist with screening	*Reception had less opportunity to direct patients to do the screening during those busier times, because they can’t get away from the phone (PM-15)*
Sustainability	
Adaptations	*I think if it was adapted in a different way to make it flow more easily. Obviously minimal intervention from reception staff (R-16)* *It would be hard but we would adapt (R-2)*
Factors impacting sustainability	*We have a diverse demographic, lots of socially disadvantaged people. The North Shore might be more tech savvy—lots of people here do not even have a phone (PM-19)* *One of the staff took it upon herself to [help patients]—we’ve got enough staff to do that (PM-17)*

GP = general practitioner; PM = practice manager; R = receptionist.

### Staff interviews

Staff interviews elicited several themes. Overall, GPs liked the self-screening concept and the improved accuracy over pulse palpation, and found it fitted in well with their workflow. Receptionists were also supportive of screening but found challenges with their workflow, as many patients required assistance with screening.

#### Acceptability of the concept

*“A good idea”:* Almost all interviewees indicated that the concept of self-screening was “a good idea.” GPs were particularly positive about the identification of cases of AF (or potential AF) that were previously unknown (GP-3, GP-10, GP-13, GP-14); the improved accuracy over pulse palpation (GP-10, GP-11, GP-13, GP-14); and the potential to reduce the burden associated with undiagnosed AF/stroke (GP-3, GP-11). Two GPs (GP-3, GP-14) mentioned the possibility for unintended negative consequences. These were increased patient stress and expense associated with follow-up investigations, and patients misunderstanding the meaning of a “normal” AF screening result and not reporting heart symptoms to their GP during a subsequent consultation.

*“A good idea, but…”:* Reception staff and practice managers also saw the value in identifying cases of AF (R-2, R-7, PM-5, PM-12, PM-15, PM-17), with 1 describing the screening station as a “life-saving machine” (R-2). However, reception staff and practice managers also tended to qualify their assessment by mentioning the impact on reception workflow (R-2, R-6, R-7, R-9, R-16, R-18, R-19, PM-5); this is discussed in more detail below.

#### Staff views on acceptability for patients

*“The patients are happy to do it”:* Many staff commented that patients appreciated having access to AF screening (R-1, R-2, R-6, R-18, PM-5, PM-12, PM-17, GP-3, GP-4, GP-10, GP-11, GP-13, GP-14). Patients were “very interested in the results” (GP-11) and appreciated the reassurance of getting a normal result (GP-10), and that they were being tested for something that they would not think of otherwise (GP-13).

*Patient refusal:* There was also discussion of patient refusal. It was not possible in the current study to determine the proportion of refusals. Staff estimates at 1 practice were about 1–2 patients a day. At another practice, staff discussed patient refusal as a major barrier. Reasons raised by staff included patients not wanting to touch the device during COVID-19 (elaborated on below); believing that it was not necessary; not wanting to know if there was something wrong; patients expecting or preferring the doctor or the nurse to do it; and wanting to be left alone while they waited for their appointment.

#### Staff views on usability of the self-screening station

*Some patients need help with screening:* Although the screening station was designed to be used independently, staff in all practices reported that a proportion of patients needed assistance with the process (R-1, R-2, R-6, R-7, R-8, R-9, R-16, R-18, R-20, PM-5, PM-12, PM-15, PM-17, PM-19, GP-3, GP-4, GP-13, GP-14). It was beyond the scope of this study to estimate this proportion; however, some staff offered estimates, ranging from “20 to 30 percent” to “every” patient. The impact this had on reception workflow is discussed in a subsequent theme.

*Not “tech-savvy”:* Staff noted that patients in the age group were not “tech savvy” (R-1, R-7, R-8, R-16, R-18, R-20, PM-5, PM-12, PM-17, PM-19, GP-13, GP-14) or that they might prefer or even expect to be shown what to do (R-2, R-6, R-7, R-18, PM-5, PM-12, GP-14). Patients from a lower socioeconomic demographic and older patients (over 70 or 75) were seen as being more likely to require or ask for assistance (R-1, R-20, PM-5, PM-12, PM-15, PM-17, PM-19, GP-13, GP-14).

#### Impact on workflow

*Minimal impact on GP workflow:* Five of the 6 GPs reported that the impact on their workflow was minimal. The time taken to review screening ECGs was not onerous, and was described by one as “worth it” (GP-13). While AF-SELF-SCREEN did not appear to substantially increase GP workload, 2 GPs (GP-3, GP-14) commented that they sometimes had to wait for a patient to finish screening, which could lead to delays for subsequent patients. Several GPs also mentioned that the screening results did not always arrive into the investigations inbox in time for them to review prior to the consultation. This issue was resolved during the evaluation period.

*Significant impact on reception workflow:* In contrast to the impact on GP workflow, impact on reception workflow was seen as significant by almost all staff (R-2, R-6, R-7, R-8, R-9, R-16, R-18, R-20, PM-5, PM-12, PM-15, PM-19, GP-3, GP-4, GP-13, GP-14). This was associated with having to come out from behind the desk and assist some patients with the screening process. The average time taken to assist a patient was estimated by staff at 2 minutes. Across the day, this time could “add up” and interfere with reception staff doing their normal duties (R-2, R-7, R-8, R-9, R-20, PM-5, PM-12, PM-15).

*Too busy to screen all patients:* Reception workflow was described as being already very busy, meaning that integrating additional work was difficult (R-2, R-6, R-7, R-18, R-20, PM-5, PM-19). While staff wanted to help patients, they prioritized their normal duties, meaning that in busy periods they might not print or offer QR codes to patients (R-2, R-8, R-9, R-16, R-18, PM-12, PM-15, PM-19), leading to reduced numbers screened, as this was an essential part of the patient onboarding process.

#### Sustainability

*Adaptations:* Three GPs suggested that AF self-screening was sustainable in its current form (GP-10) and should continue beyond the study period (GP-4, GP-11). For others, the caveat to sustainability was the issue of increased workload for reception (GP-3, GP-13, GP-14). Reception staff and practice managers also linked sustainability to the impact on reception workflow. Some receptionists believed that the program would be sustainable if it could be adapted so they did not have to assist so many patients (R-6, R-7, R-16, R-18, PM-15). A related theme was that the practice itself would adapt to accommodate the additional work, which would become easier over time (R-1, R-2, R-8, R-16, R-19, PM-17). There were also suggestions that the program might be more sustainable if COVID-19 was not a concern (see further discussion below).

*Factors impacting sustainability at a practice level:* Staff ratios at reception were mentioned by staff as a feature that could help or hinder practices from integrating AF SELF SMART, and in particular provide assistance with screening for patients who required it (R-1, R-7, R-9, PM-12, PM-17, GP-14). Patient demographics of the practice could also impact on sustainability, with suggestions that practices with a higher proportion of older patients (within the cohort) and those from lower socioeconomic backgrounds would have less success, as these patients would require more assistance (R-20, PM-12, PM-19, GP-13, GP-14).

#### Impact of COVID-19 on the study

The study was conducted during the COVID-19 pandemic, which had significant impacts on general practices in New South Wales. All participating practices worked with additional measures in place to ensure staff and patient safety, which increased the workload of reception staff. Furthermore, the vaccine rollout greatly increased reception workload. Across 3 practices, lockdowns and shifts to telehealth led to premature cessation of the program. COVID-19 was mentioned frequently by reception as a factor impacting the time they had to assist patients with screening (R-2, R-6, R-7, R-18, R-20, PM-5, PM-15, PM-19). Some staff also discussed COVID-19 with respect to the safety of the screening process, including having to frequently sanitize the station, with 2 practice managers describing staff concerns (R-8, PM-12, PM-17, PM-19).

### Patient observations

#### Appreciation for opportunistic AF screening at the practice

Most patients involved in the observations had not heard of AF. Once the purpose of the screening was explained to them, they were interested and indicated that it was a good thing to have in the practice. Many expressed gratitude for the opportunity to be screened and made comments such as “thank you so much for looking out for us oldies.” They also appeared to enjoy watching the trace and made comments such as “glad to see I’ve got a heartbeat.”

#### Difficulty with the self-screening process

The patient observations corroborated the findings from the staff interviews that some had trouble with self-screening. Very few patients were observed to complete the screening without any assistance.

Many patients had trouble identifying where to look for instructions, and appeared to make assumptions about the screening process rather than engaging with available forms of instruction, attempting to start the process by putting fingers on the ECG device; attempting to use the screening iPad as a source of instructions; and trying to complete the screen at the same time as watching the instructional video.

Patients commented that having 2 screens was “confusing” and that “1 screen would be better,” and said the instructions should be integrated into the screening process on 1 screen (“like at the airport”).

Patients also had difficulty with the QR code process. They did not understand that the QR code was on the piece of paper provided by reception when they checked in, and attempted to find a QR code on the screening station to scan using their phone. Some patients also had problems locating the ECG device or working out which fingers to put on it. Once they had located the device, some patients also had problems with getting an adequate trace.

#### The iterative design process

As discussed above, some aspects of the screening station and instructional materials were redesigned based on initial interview and patient observation findings from the first 4 practices. Patient observations suggested that some issues had been improved, but that further enhancements in design may be required. Prior to the implementation of the redesigned materials no patients (0/7) completed screening without assistance, while after redesign 27% (4/15) were able to do so. However, some patients still experienced problems engaging with the instructions, scanning the QR code, finding the ECG device, and getting a good signal (Table 2).

**Table 2:** Patient interactions with the self-screening station

Issue	Total (n = 22)	Prior to redesign (n = 7)	Following redesign (n = 15)
Required assistance finding instructions	54% (12/22)	71% (5/7)	47% (7/15)
Required assistance with QR code	45% (10/22)	71% (5/7)	33% (5/15)
Required assistance locating ECG device	23% (5/22)	14% (1/7)	27% (4/15)
Problems with poor signal	27% (6/22)	28% (2/7)	27% (4/15)
Station and ECG were awkward to use	15% (4/22)	57% (4/7)	0% (0/15)

ECG = electrocardiogram.

## Discussion

To our knowledge, this is the first study to evaluate the implementation issues of acceptability, patient usability, impact on workflow, and potential sustainability of an AF self-screening station in GP waiting rooms. GPs valued the self-screening concept and the improved accuracy over pulse palpation, and found it fitted in well with their workflow. In our study, self-screening overcame some of the barriers associated with clinician-led screening, such as consultation time constraints, and it also saw an increase in screening rates over previous Australian studies.^11^ Receptionists were supportive of the idea of self-screening, but found challenges with workflow associated with needing to assist a proportion of patients. The patient observations corroborated this finding, indicating that patients had difficulty with some aspects of the screening process.

Previous opportunistic screening approaches in general practice have focused on staff-led screening through pulse palpation or hand-held devices.^9^^,^^14^, ^15^, ^16^, ^17^ Only 1 other study, SAFE-2-SCREEN, examined patient-led screening for AF in GP waiting rooms. The SAFE-2-SCREEN study screened a large number of patients (n = 28,340, mean age = 51.9) and achieved a detection rate of 0.68%,^18^^,^^19^ which is in line with the known detection rate of 0.41% for people <65 years.^20^ The AF SELF SMART protocol differed from SAFE-2-SCREEN in 2 key ways. Firstly, SAFE-2-SCREEN relied on patients initiating screening, whereas AF SELF SMART targeted people aged ≥65 years and incorporated an SMS invitation and an invitation at the time of check-in. Secondly, SAFE-2-SCREEN did not involve any input from practice staff to complete the screening.^18^^,^^19^ In AF SELF SMART patient assistance was not explicitly written into the protocol; however, reception staff chose to help patients where required. While AF SELF SMART had a better screening rate of in-scope patients than physician- or nurse-led screening, inviting patients to screen and providing assistance to complete screening required additional work from reception staff, leading to lack of engagement with the process during busy periods. It is important to note that COVID-19 is likely to have exacerbated this impact, because it increased overall workload arising from telehealth consultations and booking/facilitating vaccination clinics.

Shifting the locus from clinician-led screening to patient self-screening is part of a wider shift toward the utilization of self-service technologies in health care. However, previous research suggests that older adults may prefer human interaction, or may experience difficulties with such technologies owing to age-related cognitive and physical decline.^21^, ^22^, ^23^, ^24^, ^25^, ^26^ Our own results support this, indicating that many older patients had problems with the self-screening process. There were also suggestions that some were unwilling to engage with it and might prefer assisted screening. This is in keeping with previous research on self-screening devices for blood pressure in GP waiting rooms.^21^^,^^25^ If upscaled, these issues could be addressed in part through careful redesign of AF self-screening kiosks so that they are optimized for use by older patients. Czaja and colleagues^27^ and others^24^^,^^28^ have noted that self-service technologies are often not optimized for older people but that careful design can make them more usable. One of the primary problems faced by patients in our study was that they did not effectively engage with the available instructional materials. Familiarity is an important aspect of successful use of self-service technologies by older people.^29^ Our findings suggest that patients were attempting to use their experience with such technologies as a guide, particularly in relation to the QR code and the attempt to use the screening iPad as a source of instruction. Patients may be more familiar with and expect interfaces in which instructions are integrated step by step into the process, and this also lessens the load on working memory, which is a function that declines with age.^27^

While careful design may go some way to reducing the need for patient assistance, there are likely to be some patients in the age group who will still require or prefer such help. Given this, a protocol that specifically allows for some level of staff involvement may be a virtue in this age group. The VITAL AF study conducted in the United States incorporated a screening device into the workflow of the clinic medical assistants, who routinely take vital signs at check-in.^30^ This role does not exist in the Australian context. One possibility is to incorporate the screening process into the reception desk. While this would place the responsibility for screening entirely on reception staff, receptionists would not have to get up from the desk to assist patients, which may be easier to integrate into workflow. However, a similar approach trialed by Orchard and colleagues^6^ found screening was not compatible with receptionists’ perceived roles, and it was noted that research-specific requirements (eg, obtaining consent) may have also had a significant impact.

An alternative possibility is a protocol that allows for load sharing by incorporating some patient self-screening, some reception-assisted screening, and some clinician-led screening. Load sharing would be facilitated by the idea that screening for AF is an all-of-practice goal, rather than being the responsibility of reception or GPs. We found that all staff saw the value of screening for AF, which is an important element in achieving this. A protocol that allowed for load sharing could have the added advantage of reducing patient refusal. Patients who refused screening at reception may be more amenable to being screened during their appointment. There was some suggestion of this in interviews, and Vassli and Farshchian^26^ have identified unwillingness to have technology replace human interaction as being a major barrier to uptake of self-service technologies in health care by older people.

Self-screening stations for AF have the potential to overcome previously identified barriers to screening in general practice. However, prior to future implementation and upscaling, certain aspects of the process need to be addressed to facilitate increased patient usability, as well as acceptability and sustainability for practices.

The key recommendations are as follows:

• Include an iterative, consumer-centered design process to optimize the design of the screening station.
• Ensure the station is set up in line with prior experiences and expectations (ie, “familiarity”).
• Incorporate real-time, step-by-step instructional materials into the screening device interface.
• Streamline the onboarding process using intuitive “familiar” technology to increase patient usability.
• Redesign the kiosk and screening interface to improve accessibility and positioning of fingers on ECG device.
• Develop a protocol that allows for some assistance to be provided and allows for load sharing of screening responsibility across practice staff.

### Limitations

The current study was exploratory and qualitative and relies on staff perspectives and patient observations. As such, it was unable to determine the actual percentage of patients who required assistance from reception, or how many were offered screening by reception at each practice, and who refused self-screening when offered. We were also unable to formally test the impact of age and socioeconomic background on ability to use the station. Further research could address this, along with other factors such as being from a non-English-speaking background. Although our iterative design allowed us to make some modifications during the course of the research, time and technical constraints prevented implementation of all ideal modifications. It was beyond the scope of the present study to completely redesign the screening station, including incorporation of instructions and screening process into 1 device.

## Conclusion

Self-screening for AF was seen as valuable by all staff; however, the increased workload placed on reception by the current screening prototype was a barrier that would need to be addressed if AF self-screening was implemented more widely. Improvements to the instructional materials and the patient interface of the screening station may alleviate this issue. Further process improvements could include a modified protocol that allowed for reception- and clinician-assisted screening for patients who were unable or unwilling to undertake self-screening. Further research is required to develop this approach and assess its acceptability and effectiveness for upscaling in general practice in Australia.

## Supplementary data

Supplement 1_Iterative redesign process

Supplement 2_Interview schedule Clean

Supplement 3_Coding table
