# Supplementary material for: Staff acceptability and patient usability of a self-screening kiosk for atrial fibrillation in general practice waiting rooms
Source: Cardiovasc Digit Health J. 2022 Aug 4;3(5):212–9. doi: 10.1016/j.cvdhj.2022.07.073 (PMC9596310; doi:10.1016/j.cvdhj.2022.07.073)
Supplement: Supplement 1_Iterative redesign process [file mmc1.docx]

**SUPPLEMENT 1**

**Iterative redesign process**

The initial screening station consisted of a table with an iPad, a KardiaMobile single lead ECG device inserted below the iPad, instructional materials printed on the table, and an additional iPad with video instructions. The written instructions were incorporated into the table top itself and included text and icons representing each step of the screening process. The video was looped so that it was playing constantly. The screening iPad was located to the right of centre, and the written instructions and instructional iPad was located on the left side of the table.

After analysis of the initial staff interviews and patient observations, the following changes were made.

- The written instructions were relocated to a plastic holder so that they were vertical not horizontal.
- The written instructions were reworked to include photographic images representing each stage of the process, to accompany the text.
- The screening iPad was relocated so as to be more central.
- The video was changed to be user-initiated rather than looped.
