# Supplementary material for: Staff acceptability and patient usability of a self-screening kiosk for atrial fibrillation in general practice waiting rooms
Source: Cardiovasc Digit Health J. 2022 Aug 4;3(5):212–9. doi: 10.1016/j.cvdhj.2022.07.073 (PMC9596310; doi:10.1016/j.cvdhj.2022.07.073)
Supplement: Supplement 2_Interview schedule Clean [file mmc2.docx]

# SUPPLEMENT 2: SEMI-STRUCTURED INTERVIEW QUESTIONS

# RECEPTION STAFF

1. Overall, how satisfied were you with the self-screening process?
2. Were there any issues with the generation of text message prompts for patients?
3. Did patients ask you about AF screening? Did you feel you were able to adequately answer their questions? If not, why did you feel you were unable to address their questions?
4. How do you think the procedure of performing their own screening went for patients? Did they experience any difficulties with the device? Did they experience issues with the screen prompts?
5. How do you feel patients responded to the self-screening?
6. Did the self-screening impact on your workflow in any way?
7. Were there any issues with dealing with abnormal results?
8. Can you see a sustainable role for opportunistic self-screening for AF among older adults using a self-screening kiosk in general practices? Why or why not?
9. What are your recommendations for improving the self-screening process to make it more suitable for opportunistic screening in the general practice environment?
10. Are there any other issues not covered that you would like to talk about?

**Thank you for taking the time to complete this interview.**

**We truly value the information you have provided.**

# PRACTICE MANAGERS

1. How satisfied were you with the screening program overall?
2. Do you feel that practice staff received sufficient training and ongoing support? If not, what extra training or support would have been useful for them?
3. Were there any aspects of the screening process that you did not like or were difficult for your practice? If so, can you describe these in more detail?
4. Was there a cost to providing the screening to your practice? Do you think the financial assistance provided by the research team was sufficient to cover the cost?
5. In what ways did the screening program impact on the workflow of the practice? Do you have any comments on the amount of work involved for you, and if so, what are they?
6. Do you think that opportunistic self-screening for AF in general practice using the screening kiosk is a feasible method of screening for patients > 65 years of age? Why or why not?
7. Do you have any suggestions on how the process could be improved?
8. Are there any other issues not covered that you would like to talk about?

**Thank you for taking the time to complete this survey.**

**We truly value the information you have provided.**

# GENERAL PRACTITIONERS

1. How satisfied were you with the self-screening program overall?
2. What aspects were useful for your patients and your practice?
3. Do you normally routinely screen for AF in your patients aged > 65 years? If so, when and how often would you screen them? If not, why are you not performing regular screening?
4. How did your patients respond to the self-screening program?
5. Did the self-screening program add any more time to your consultations?
6. Did your practice have a clear protocol for dealing with abnormal results?
7. Was there anything you didn’t like or found difficult with the self-screening?
8. Did the self-screening program interfere with workflow in the practice?
9. Can you see a sustainable role of opportunistic self-screening for AF among older adults using a self-screening kiosk in general practices? Why or why not?
10. What are your recommendations for improving the self-screening process to make it more suitable for opportunistic screening in the general practice environment?
11. As a result of the study, did you change the management of any patient with known atrial fibrillation? Did you change management regarding oral anticoagulation?
12. Are there any other issues not covered that you would like to talk about?

**Thank you for taking the time to complete this interview.**

**We truly value the information you have provided.**

**ADDITIONAL COVID INTERVIEW QUESTIONS**

**GP questions**

-How did Covid-19 impact your practice/workflow?

-What do you think of self-screening during Covid-19?

-In what ways do you think Covid-19 has impacted on patient self-screening, and are there any recommendations for improvement for patient self-screening?

**Reception/PM questions**

-How did Covid-19 impact your workflow and patient traffic?

-Did patients decline screening out of fear of Covid-19? If yes, what did they say?
