# Supplementary material for: Staff acceptability and patient usability of a self-screening kiosk for atrial fibrillation in general practice waiting rooms
Source: Cardiovasc Digit Health J. 2022 Aug 4;3(5):212–9. doi: 10.1016/j.cvdhj.2022.07.073 (PMC9596310; doi:10.1016/j.cvdhj.2022.07.073)
Supplement: Supplement 3_Coding table [file mmc3.docx]

**Code book**

| **Code name** | **Content** |
| --- | --- |
| **Acceptability of the concept** |  |
| *“A good idea”* | References to the initiative being a good idea, valuable, necessary, important. Include reasons (e.g. it reduces burden on the healthcare system; it could save someone’s life). Also include qualified references (e.g. it is important but it takes up too much time). |
| *Detecting cases* | Any mention of how the initiative resulted in a case or suspected case of AF being detected. |
| *More accurate* | Include references to the results being more accurate than other methods. |
| *Negative outcomes* | References to the potential for negative outcomes *for the patient.* Do not code here if negative outcome is for staff (e.g. increased workload). |
| **Staff perspectives on acceptability and useability for patients** |  |
| *“Patients are happy to do it”* | References to patients enjoying/liking/appreciating the initiative. Include reasons (e.g. they like knowing that there is nothing wrong with their hearts) |
| *Patient refusal* | References to patients refusing to participate in the initiative. Include reasons (e.g. they don’t want to touch the machine). Include estimates of how many patients refuse. |
| *Some patients need help with screening* | Any references to the patient needing assistance, or asking for assistance. Consider also coding in *impact on workflow*. |
| *How many patients need help* | References to, or estimates of the number/percentage/proportion of patients who need assistance with screening |
| *“Our patients are not tech-savvy”* | References to patients being unable to self-screen because they lack computer literacy. Include reasons (e.g. that generation are not very good with computers). Code here and in ‘*patients need assistance with screening’* |
| *The patients want/expect to be helped* | References to patients wanting or expecting to be assisted. May include commentary on why such as age or other demographic variables |
| **Impact on workflow** |  |
| *Impact on GP workflow* | References to the impact on GP workflow (e.g. it only takes a minute). Include references to direct and indirect impact (e.g. time taken to review a patients screening results in the consult (direct) and being kept waiting because a patient is self-screening (indirect)). Do not include references to GP awareness of impact on reception workflow. |
| *Impact on reception workflow* | Any reference to the impact the study has on reception workflow. Include awareness of impact on other staff (e.g. it doesn’t impact me but I know it is difficult for reception staff). Include references to the study interfering with normal duties. |
| *Time taken to assist patients* | Reference to the time it takes to assist patients with self-screening (e.g. about 2 minutes). |
| *Too busy to screen patients* | References to reception being very busy in general. References to reception not having time to assist patients or to some patients not being screened in busy periods or because reception staff are too busy. Also code in *Covid-19* if mentions Covid-19*.* |
| **Sustainability** |  |
| *Like it to continue* | References to a desire for the initiative to continue beyond the study period. |
| *Sustainable with adaptions* | References to the study being sustainable if impact on reception is addressed, or sustainable if adaptions are made to reduce need for reception to assist patients. |
| *“We would adapt”* | References to the process becoming easier over time, or becoming part of what the practice would do going forward. May include qualifications (e.g. we would adapt but we would not screen every patient). |
| *Factors impacting sustainability* | References to specific aspects of the practice that might be relevant to the sustainability of the study. These include staff ratios; and patient demographics (consider also coding in *the patients can’t do it by themselves*). |
| *Covid-19* | References to the impact of Covid-19 on the workload at the practice, and/or on the sustainability of the initiative (e.g. it would have been easier if it wasn’t for Covid). *Consider also coding in impact on workflow* and *too busy to help patients*. |
